# Effects of Turmeric and Rosemary Extract on Oxidative Stress Markers in Goats

**DOI:** 10.3390/ani15030369

**Published:** 2025-01-27

**Authors:** Daria M. Urbańska, Natalia Kurhaluk, Halyna Tkaczenko, Karolina Rutkowska, Ewelina Kawecka-Grochocka, Paulina Brzozowska, Michał Czopowicz, Marcin Mickiewicz, Jarosław Kaba, Emilia Bagnicka

**Affiliations:** 1 Department of Biotechnology and Nutrigenomics, Institute of Genetics and Animal Biotechnology Polish Academy of Sciences, Postepu. 36A, 05-552 Jastrzebiec, Poland; 2 Department of Biology, Institute of Biology, Pomeranian University in Słupsk, 76-200 Słupsk, Poland; natalia.kurhaluk@upsl.edu.pl (N.K.); halyna.tkachenko@upsl.edu.pl (H.T.); 3 Department of Medical Genetics, Medical University of Warsaw, Pawińskiego 3c, 02-106 Warsaw, Poland; karolina.rutkowska@wum.edu.pl; 4 Division of Veterinary Epidemiology and Economics, Institute of Veterinary Medicine, Warsaw University of Life Sciences-SGGW, Nowoursynowska 159c, 02-776 Warsaw, Poland; michal_czopowicz@sggw.edu.pl (M.C.); marcin_mickiewicz@sggw.edu.pl (M.M.); jaroslaw_kaba@sggw.edu.pl (J.K.)

**Keywords:** oxidative stress, male goat, polyphenols

## Abstract

Background: Due to their versatility, goats are becoming popular as livestock on commercial farms. To obtain a high productivity, the maintenance of homeostasis in these organisms is needed. Some supplements that could help improve their health state have been tested recently. The study examines the effects of a turmeric–rosemary extract mixture on the antioxidant status of the blood of young castrated bucks. Results: Supplementation lowered lipoperoxidation and increased TAC, probably due to the improved neutralization of reactive oxygen species (ROS) by enhanced antioxidant pathways. Despite lowering lipid peroxidation, these products can increase protein oxidation. Thus, while enhancing lipid defense mechanisms may reduce lipid peroxidation, it may also increase the level of OMP, probably due to the accumulation of lipid peroxidation metabolites. The activities of SOD, GPx, and GR remained unchanged, whereas CAT activity decreased. CP, ALT, and AST remained stable, suggesting no adverse effects on key markers of liver function. Conclusions: The high antioxidant potential observed after supplementation indicates that the turmeric–rosemary extract mixture elicited a favorable biochemical response; the supplemented compounds effectively enhanced antioxidant defenses (by improving the neutralization of ROS) without negatively affecting liver enzyme activities. Turmeric–rosemary dry extract supplementation can help with homeostasis maintenance in goat organisms and, consequently, improve their productivity.

## Simple Summary

Animal welfare is a primary concern within the development of livestock farming and animal health is one of the major issues it refers to. It is possible for organisms to be protected against many diseases due to the proper functioning of the immune system. Oxidative stress can cause damage to DNA, proteins, and lipids in cells. Consequently, it can lead to cell and tissue damage. Natural and easy-to-produce supplements are increasingly used in farming. We hypothesized that polyphenols influence oxidative stress. Therefore, the aim of our study was to analyze the effect of supplementation with a dried turmeric and rosemary extract mixture on the oxidative stress markers in the blood serum of young castrated bucks of the Polish White Improved (PWI) breed. We suggest that supplementation effectively enhanced antioxidant defenses without adversely affecting liver enzyme activities: a favorable biochemical response. However, further studies are needed to determine the effect of different doses of the dry turmeric–rosemary mixture.

## 1. Introduction

Nutrition plays an important role in ensuring the health and productivity of goats on farms [1]. Diet composition has a significant impact not only on animal health, but also on the quality of their products, such as milk and meat. An appropriate diet containing essential nutrients can significantly improve their growth, development, and reproductive health, resulting in better breeding performance, fewer health problems, and greater productivity [1,2,3]. A key goal for ensuring goat health, productivity, and sustainable agriculture is devising effective forage enrichment. Such research not only responds to current agricultural challenges, but also contributes to the development of more sustainable farming practices worldwide [4]. The drive to develop effective feed supplements, for example, based on natural plant extracts, is critical to improving overall animal health and production efficiency [5,6]. Despite advances in research into these extracts and their potential benefits for goats, further research is still needed to fully understand the mechanisms of action of feed ingredients and their optimal doses to maximize their beneficial effects on animal health and productivity [4].

One area of particular interest is the supplementation of goats with different blends of plant extracts, as this may strengthen the immune system, reduce inflammation, and protect against oxidative stress; thus, supplementation can result in a better production performance, increased milk production, and weight gain [7]. The role of oxidative stress is mainly studied in veterinary medicine with a focus on mastitis, pneumonia, and retained placenta. However, it participates in the pathophysiology of numerous diseases. Discovering an effective way to protect the body against oxidative stress may improve the productivity and health of small ruminants [8]. In addition, natural supplements may help reduce the need for antibiotics, which is a key consideration in the face of increasing bacterial resistance. Furthermore, this research can contribute to the development of more sustainable and environmentally friendly farming practices, benefiting farmers, consumers, and the environment alike [9].

When choosing a plant as a dietary supplement, species from the *Lamiaceae* family have aroused considerable interest due to the presence of rosmarinic acid [10]. Rosmarinic acid has a wide range of beneficial properties, including anti-inflammatory [11], antimutagenic [12], antitumor, and antiproliferative activities [13]. It has also demonstrated various anti-cyclooxygenase effects [14], anti-allergy properties [15], and antidepressant activity [16]. Rosmarinic acid has also been found to have antiviral activity and act as a potent natural antioxidant [17], potentially protecting against conditions induced by free radicals.

Both clinical studies and veterinary medicine have found turmeric (*Curcuma longa*) to have various pro-health effects which have been attributed to its numerous constituents, particularly major curcuminoids [18]. The health benefits of turmeric are believed to revolve around curcumin, a lipophilic polyphenolic compound with an orange-yellow hue that is derived from the rhizome. Indeed, recent research has found curcumin to have various antioxidant, anti-inflammatory, and anti-cancer properties [19], which may support its role in the prevention and treatment of inter alia cancer, autoimmune disorders, neurological conditions, and cardiovascular disease [20].

Various studies indicate that supplementation with turmeric (*Curcuma longa*) or rosemary (*Rosmarinus officinalis*) extracts have multiple beneficial effects on the health, performance, and quality of goat products, which may have important implications for breeding practices and food production [21,22].

However, most of these experiments dealt only with one supplement, turmeric or rosemary, and information on the influence of both uses together is limited. As this herb and spice are used in many human cuisines together, and goats can be used as an animal model for humans [23], the results of experiments using a mixture of the extracts of both plants will be very useful. Before now, several such experiments have been conducted by our team. A study of the effect of curcumin and rosemary mixture supplementation in young castrated bucks [24] showed that supplementation may modulate the immune response by influencing the expression of the genes controlling certain cytokines, including *IL-1β*, *IL-6*, and *TNF-α*, in the liver. Similarly, the *Curcuma longa* and *Rosmarinus officinalis* extracts altered the genetic expression of acute phase proteins, cathelicidin, defensin, and cytolytic proteins in the liver, particularly those related to inflammatory responses and defense mechanisms; this may suggest that adding turmeric and rosemary to animal diets offers potential health benefits [23]. The studied mixture also had beneficial effects on weight gain and improved meat quality, especially in terms of fat content, in young castrated PWI bucks [25].

In summary, using rosemary and turmeric has some benefits on the production traits of small ruminants. However, the influence of such supplementation on oxidative stress markers must be carefully checked before commercial use. As relatively few studies have examined the effect of supplementation on antioxidant balance in livestock, this has been chosen as the focus of our investigation, particularly because oxidative stress was not studied after the supplementation with a mixture of both plants. The findings should provide a clearer picture of how the use of the applied supplements affects the antioxidant status of goat blood. Not only will the results be valuable for scientific research, but they may have considerable practical applications in medicine and veterinary science.

The aim of the study is to determine the effect of turmeric–rosemary extract on various aspects of the antioxidant status of the blood of young PWI bucks: (1) the levels of lipid peroxidation and oxidative modification of proteins (OMPs) in the serum; (2) blood total antioxidant capacity (TAC); (3) antioxidant enzyme activity, *viz.* superoxide dismutase (SOD), catalase (CAT), glutathione peroxidase (GPx), and glutathione reductase (GR); (4) and selected biochemical parameters including alanine (ALT) and aspartate (AST) aminotransferase activities and ceruloplasmin (CP) levels. These enzymes and parameters are essential for understanding the body’s defense mechanisms in response to oxidative stress.

## 2. Materials and Methods

### 2.1. Experimental Groups

The experiments were carried out on 20 young PWI castrated bucks. The mean weight of the bucks was 28.80 kg (±4.93 kg) at the beginning of the experiment and were approximately eight months old. The bucks were castrated at three months of age. Using the internet tool biomath.info (http://biomath.info/power/index.html; accessed on 11 January 2025) with assumptions for alpha: Prob (reject H_0_ when H_0_ is true) = 0.01, and Power: prob (reject H_0_ when H_1_ is true) = 0.90, we selected 20 castrated bucks for the study. The animals were divided into two groups: control (N = 10) and treated (N = 10). All animals were kept under the same conditions and fed according to a system developed by the Institut National de la Recherche Agronomique (INRA) in France, adapted to the nutritional value of the feed used in Poland [26]. The basal diet was the same for both groups. Detailed information on the diets was presented in [25]. As goats in temperate climates are seasonal animals, all offspring born in a given year are of a very similar age and reach similar body weight at approximately the same time. As all of them were affected by the same factors at the same time, seasonal and environmental variables were not taken into account.

The bucks in the treated group were supplemented with 1.6 g/day of a dry extract mixture of turmeric and rosemary in a ratio of 896:19 (Selko^®^ AOmix, Trouw Nutrition Polska sp. z o.o., Grodzisk Mazowiecki, Poland). The extract used is a commercial supplement and the ratio of Rosmarinus officinalis to Curcuma longa was established by the manufacturer. The producer also recommended dosing the supplement for young castrated bucks based on their previous experiences in dairy cattle and dairy goat breeding. The supplement was packaged in starch capsules and administered orally before the morning feed. Detailed information on dietary supplementation, growth performance, meat quality, lipid metabolism, and the immune system gene expression of the two groups of animals has been reported previously [23,24,25].

### 2.2. Samples

Samples were taken once after the end of the experiment, i.e., on day 124 from the start of supplementation. The blood samples were collected from the jugular vein immediately after sacrifice from all animals on the same day. The collection was performed using separate tubes containing a coagulation activator for each animal (Sarstedt AG & Co., Nümbrecht, Germany). After two hours, the blood samples were centrifuged for serum collection. The blood serum was stored at −20 °C for further analysis. Each biochemical analysis was conducted in duplicate.

### 2.3. Biochemical Assays

TBARS assay for lipid peroxidation. Lipid peroxidation was determined using the malonic dialdehyde (MDA) method proposed by Buege and Aust [27], based on measuring the concentration of 2-thiobarbituric acid reactive substances (TBARSs). Results were expressed as nmol per mL.

The protein carbonyl derivative assay. The OMPs level was determined using the protein carbonyl derivative assay. The resulting carbonyl derivatives of amino acids were reacted with 2,4-dinitrophenylhydrazine (DNFH) according to Reznick and Packer [28], as modified by [29]. Carbonyl groups were quantified spectrophotometrically at 370 nm and 430 nm (for aldehyde derivatives, AD OMP, and ketone derivatives, KD OMP, respectively) and the results were expressed as nmol per mL.

Superoxide dismutase activity. SOD activity was determined according to [30]. In brief, the ability of SOD to dismutate superoxide generated during the auto-oxidation of quercetin was measured in an alkaline medium (pH 10.0). The resulting activity was expressed as units of SOD per mL.

Catalase activity. CAT activity was determined by measuring the reduction of H_2_O_2_ in the reaction mixture at a wavelength of 410 nm, as described by [31]. One unit of CAT activity was defined as the amount of enzyme required to reduce 1 μmol H_2_O_2_ per minute per mL.

Glutathione reductase activity. The activity of GR was measured according to [32] with a few modifications. GR activity was expressed as nmol NADPH2 per minute per mL.

Glutathione peroxidase activity. GPx activity was determined by observing non-enzymatic consumption of the reacting substrate GSH at 412 nm absorbance after incubation with 5,5-dithiobis-2-nitrobenzoic acid (DTNB) according to [33]. The resultant GPx activity was expressed as μmol GSH per minute per mL.

Total antioxidant capacity. TAC was assessed by quantification of TBARS after the oxidation of Tween-80 [34]. This level was determined spectrophotometrically at 532 nm. Inhibiting Fe2+/ascorbate-induced oxidation of Tween-80 resulted in decreased TBARS levels. The TAC content in the sample (%) was calculated relative to blank absorbance.

Ceruloplasmin (CP) assays. CP was determined using the Ravin method in 0.4 M sodium acetate buffer (pH 5.5) and 0.5% p-phenylenediamine at 540 nm according Kamyshnikov [35]. The results were expressed as mg per %.

### 2.4. Statistical Analysis

Data were analyzed with Statistica software 13.3 (Tibco Software Inc., Palo Alto, CA, USA). Data for both the control and supplementation groups were expressed as the mean and standard deviation (SD). As only the mean values of two groups (the control and treated groups) were compared, the independent samples *t*-test was used. The other effects such as age, body weight, and breed were similar for all animals, and the environment was the same; therefore, they were not considered in the statistical analysis. The castrated bucks were descendants of different mothers but only two bucks; within each group, one buck had six offspring while the second one had four offspring. The relationships between different biochemical parameters within each group, i.e., the strength and direction of the linear relationships between pairs of variables, were determined with Pearson’s correlation coefficients (r); these were calculated separately for pairs of parameters within each group. The significance of the correlations was assessed using the corresponding *p*-values, with *p* < 0.05 indicating a significant correlation.

## 3. Results

### 3.1. Oxidative Stress Biomarkers

**Figure 1:**
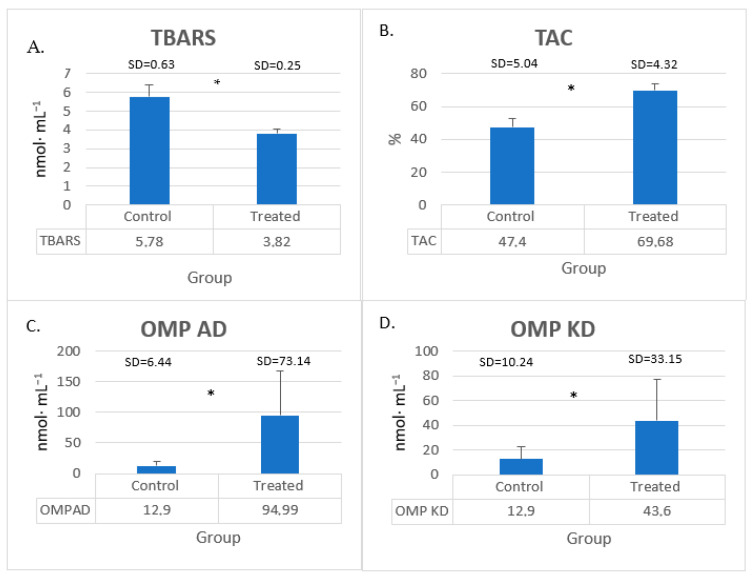
Young bucks’ blood serum levels of (**A**) TBARS, (**B**) TAC, (**C**) OMP AD, and (**D**) OMP KD after turmeric–rosemary supplementation. TRE—treated group; TBARS—2-thiobarbituric acid reactive substances (nmol∙mL^−1^); TAC—total antioxidant capacity (%); OMP AD—aldehyde derivatives of oxidatively modified proteins (nmol∙mL^−1^); OMP KD—ketone derivatives of oxidatively modified proteins (nmol∙mL^−1^). Data are expressed as mean ± SD (N = 10). * differences *p ≤* 0.05 between control and TRE group.

Our present findings indicate a significant decrease in the TBARS level in the young PWI castrated bucks after turmeric–rosemary extract supplementation (Figure 1A), accompanied by a significant increase in the total antioxidant capacity (TAC) (Figure 1B). In addition, supplementation was associated with a significant increase in aldehydic derivatives of oxidatively modified proteins (OMP AD) (Figure 1C) and ketonic derivatives of oxidatively modified proteins (OMP KD) (Figure 1D) levels in the serum.

### 3.2. Antioxidant Enzymes

**Figure 2:**
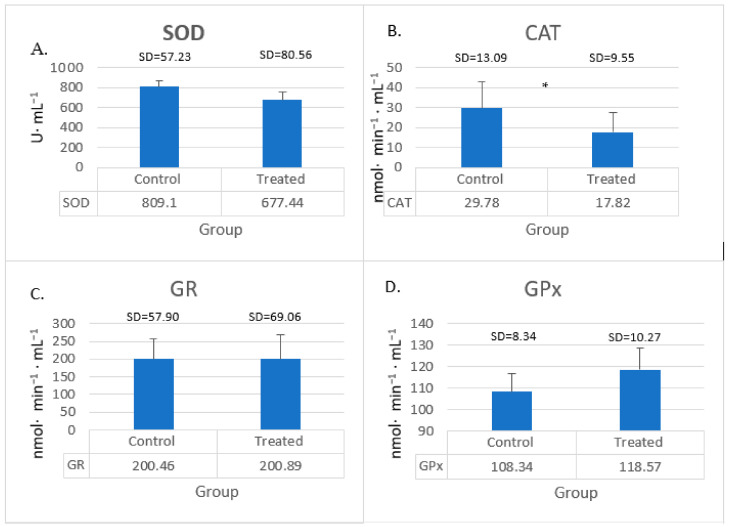
Young bucks’ blood serum levels of (**A**) SOD, (**B**) CAT, (**C**) GR, and (**D**) GPx after turmeric–rosemary supplementation. TRE—treated group; SOD—superoxide dismutase; CAT—catalase; GR—glutathione reductase; GPx—glutathione peroxidase. Data are expressed as mean ± SD (N = 10). * Differences, *p ≤* 0.05, between control and TRE group.

The effect of supplementation on the activity of the antioxidant enzymes SOD (Figure 2A), CAT (Figure 2B). GR (Figure 2C) and GPx (Figure 2D) are given; these are responsible for neutralizing ROS, which can cause lipid peroxidation, oxidative modification of proteins, and DNA damage. Interestingly, while SOD, GPx, and GR (Figure 2A,C,D) activity did not change after supplementation, CAT activity was significantly reduced (Figure 2B).

### 3.3. Biochemical Parameters

**Figure 3:**
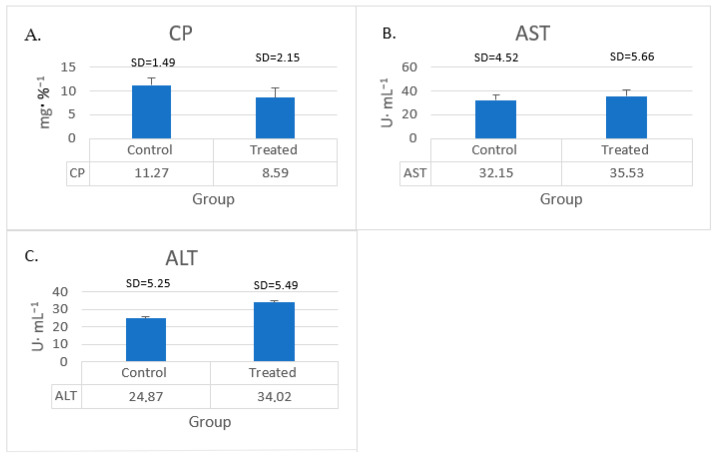
Young bucks’ blood serum levels of (**A**) CP, (**B**) AST, and (**C**) ALT after turmeric–rosemary supplementation. TRE—treated group; CP—ceruloplasmin; AST—aspartate aminotransferase; ALT—alanine aminotransferase. Data are expressed as mean ± SD (N = 10).

The results of our analyses of CP, ALT, and AST are shown in Figure 3. No statistically significant changes in the levels of all these parameters were observed in our study.

### 3.4. Correlations

Correlations (Table 1) between OMP AD and AST (r = −0.652, *p* = 0.030), GPx and AST (r = 0.764, *p* = 0.006), CP and GR (r = 0.819, *p* = 0.002), and TBARS and ALT (r = 0.614, *p* = 0.004) were noted in the control group. In the treated group, we noted a dependence between TAC and OMP AD (r = −0.788, *p* = 0.004) and OMP KD (r = 0.744, *p* = 0.004), accordingly, and between OMP KD and GR (r = 0.626, *p* = 0.040). As expected, ALT and AST were highly correlated in both groups.

## 4. Discussion

Our analyses yielded valuable data regarding the effects of the tested turmeric and rosemary mixture on health and antioxidant status of blood. Not only is this important for future research but it also has practical implications in veterinary science. It is important to understand the effect of supplementation on the antioxidant status of animals [36]. Lipid peroxidation and oxidative protein modifications are critical indicators of oxidative stress and defenses against it. It is well known that lipid peroxidation can damage cell membranes, resulting in various diseases and inflammatory conditions; it can also be used to indicate the level of oxidative stress in blood serum by measuring the level of TBARS. We noted a decreased TBARS content in the blood serum of castrated bucks in the treated group. Kim et al. [37] studied the influence of two levels of turmeric supplementation (2.5%—TPA group; 5%—TPB group), along with elevated fat and cholesterol levels, on oxidative stress in the liver of rats, comparing a fat- and cholesterol-rich diet without turmeric (HF group) and a standard diet (N group). The TBARS values were the lowest in the N group and the highest in the HF group. This suggests that powder turmeric supplementation may reduce oxidative damage in the livers of rats fed a high-fat and -cholesterol diets by activating the antioxidative defense systems. Nieto et al. [38] also found reduced TBARS and rancid odor (RO) scores in the stored meat of lambs whose mothers were fed with a distillate from rosemary leaf during pregnancy. Moreover, the highest RO scores were observed in the samples with the highest lipid oxidation. Thus, the results suggest the effectiveness of rosemary in protecting tissues against oxidation due to reactive oxygen species. We can therefore assume that the rosemary–turmeric dry mixture activates the antioxidative defense system in castrated bucks from the treated group in the presented study. Moreover, our findings indicate that supplementation with compounds known to have antioxidant properties resulted in a decrease in lipid peroxidation and an increase in TAC. Similarly to our results, El-Sherbiny et al. [39] noted increased TAC levels in the blood of mid-pregnancy goats supplemented with curcumin and olive oil nanocomposite. This can be attributed to the activation of antioxidant pathways and the improved neutralization of ROS and reactive nitrogen species (RNS). Indeed, previous studies have noted that the antioxidants present in turmeric and rosemary extracts enhance natural defense mechanisms, leading to a reduction in oxidative damage to lipids [21,22]. This increase in the levels of OMP AD and OMP KD may result from complex molecular processes such as the induction of protein oxidation and lipid peroxidation, the activation of oxidative stress-related signaling pathways, and the regulation of antioxidant pathways. The reduction in lipoperoxidation and increase in TAC observed after supplementation may be due to the activation of antioxidant pathways and the improved neutralization of ROS. Similar to our results, Ferenchuk et al. [40] noted higher levels of OMP370 and OMP340 in rats with nephropathy compared to a control group. The increase in OMPs is one of the pathogenetic links in developing pathological conditions due to oxidative stress. Verveha et al. [41] suggest that increased OMP370 and OMP430 levels are evidence of the intensification of the processes of oxidative modification of proteins and a notable increase in OMP370 and OMP430 levels could be caused by insufficient enzymatic and non-enzymatic links in the antioxidant system. Therefore, the correlation between and increase in OMPs may indicate that the rosemary–turmeric dry mixture caused oxidative stress in the liver; however, it was not strong enough to stimulate other antioxidant markers.

Turmeric is known to contain a range of carbohydrates, essential oils, fatty acids, curcuminoids (curcumin, demethoxycurcumin, and bisdemethoxycurcumin), polypeptides such as turmeric, sugars, proteins and resins [42]. The active ingredient is believed to be curcumin, which is extracted from the powder of the dried rhizomes of *Curcuma longa* and has a variety of chemical, biological, and pharmacological properties. In addition to its antioxidant potential, curcumin has been found to have various anti-inflammatory, antimicrobial, antifibrotic, and immunomodulatory properties and appears to be effective against cancer and diabetes [18,43]. Similar properties have also been attributed to demethoxycurcumin and bisdemethoxycurcumin [19], and the turmerone and zingiberene in turmeric essential oils, have also demonstrated antibacterial, anti-inflammatory, and antioxidant properties [19,20]. As such, turmeric appears to be valuable not only as a spice but also as a dietary supplement.

Rosemary is an evergreen perennial medicinal plant of the *Lamiaceae* family. It is used in medicine, aromatherapy, perfumery, and as a natural preservative in the food and cosmetics industries [44]. The leaves, shoots, and whole plant extract are valued as a functional food (antioxidant) and herbal nutraceutical. Rosemary has a range of medicinal properties, which have been attributed to its complement of bioactive compounds [45]. In particular, it produces various phenolic acids, such as rosmarinic acid, with powerful antioxidant, anti-inflammatory, antiviral, and antibacterial properties. It is also a potent source of terpenoids such as carnosic acid, carnosol, and urosole: carnosic acid is a potent antioxidant and anti-inflammatory compound, carnosol has antioxidant, anti-cancer, and anti-inflammatory activity, and urosole has antibacterial and anti-inflammatory properties. Its essential oils such as 1,8-cyneol (eucalyptol) and camphor also have antibacterial, antifungal, and anti-inflammatory properties, making rosemary a popular ingredient in natural medicines and cosmetics [10]. In addition, these compounds are all effective scavengers of ROS and RNS. Their presence thus increases antioxidant capacities and protects cellular components from oxidative stress [42].

Our findings also indicate the elevated oxidative modification of proteins after supplementation. This can be explained by the increased production of aldehyde and ketone derivatives as a result of lipid peroxidation. These reactive aldehyde and ketone products can interact with and modify protein structures, thus potentially altering their biological functions. Despite lowering lipid peroxidation, these products can increase protein oxidation. This suggests a complex interplay between lipid and protein oxidation, where increased lipid protection may simultaneously increase protein oxidative modifications due to the abundance of lipid peroxidation by-products.

TAC analysis is an effective measure of the ability of an organism to neutralize free radicals, such as ROS and RNS, and various other oxidative substances that can cause cell and tissue damage. TAC is used to estimate the complex sum of various components, including antioxidant enzymes (such as SOD, CAT, GPx and GR) and low-molecular-weight antioxidants (such as vitamins C and E, glutathione) [46]. In addition, TAC is a crucial parameter in animal studies and in the evaluation of new feed additives, because it assesses the organism’s ability to defend itself against oxidative stress induced by various environmental factors, including diet and housing conditions [47,48]. Supplementation with new feed additives, such as plant extracts, containing high concentrations of antioxidants can influence the TAC of the livestock [46]; this parameter can also be used to determine the effectiveness of supplementation and potential health benefits. Our present findings indicate a decrease in lipid peroxidation and an increase in TAC following the administration of antioxidant compounds. This may be due to the activation of antioxidant pathways by the bioactive components present in turmeric and rosemary; these are thought to enhance the defense against oxidative stress by increasing antioxidant enzyme activity and boosting endogenous antioxidant levels. This reduction in oxidative lipid damage contributes to an overall improvement in oxidative balance.

Our findings also indicate significant changes in CAT activity, as well as in important biochemical markers, i.e., TBARS, TAC, OMP AD, and OMP KD levels. These parameters provide insight into the mechanisms by which the body responds to oxidative stress. Antioxidant enzymes have key roles in maintaining oxidative homeostasis and protecting cells from damage caused by ROS, thus ensuring cellular health and preventing oxidative stress-related diseases. This reduction may be due to various mechanisms, one of which being ROS formation by the active compounds in turmeric and rosemary; these can modify proteins, including CAT. However, further studies are needed to better understand the specific interactions between these compounds and CAT and their effects on the antioxidant functions in PWI bucks. CP serves as the primary transporter of copper, which is essential for many vital enzymatic redox reactions, while ALT and AST play key roles in amino acid metabolism. It was known that elevated serum levels of ALT and AST often indicate liver and myocardial cell damage, with ALT being more specific for liver damage and AST for myocardial damage. The lack of any changes in ALT and AST levels suggest the absence of tissue damage, indicating that supplementation had a positive effect on health, with no adverse effects associated with liver function or oxidative stress, and a high antioxidant potential. This suggests that the supplemented compounds effectively enhanced antioxidant defenses without adversely affecting liver enzyme activities, reflecting a favorable biochemical response.

The correlation analysis confirmed that correlation dependencies differ between the experimental and the control groups. However, as both AST and ALT are markers of liver cell damage, thus the high and positive connection between them was expected [49]. There are no results presented in the available literature regarding the relationship between the levels of OMPs, TAC, and antioxidant enzymes activity in goat organs or tissues. However, our findings suggest a complex interplay between lipid protection and protein oxidation processes.

To avoid confounding between groups and treatments and to keep the bias of the results as low as possible, we built very analogous groups; we used animals of the same breed of very similar age and body weight, and they were maintained in the same environmental conditions. To have genetically similar groups we used the same number of offspring of two bucks in both groups (six and four in each group, respectively). As a whole, our findings demonstrate that while enhancing lipid defense mechanisms may reduce lipid peroxidation, it may also increase the level of oxidative modification in proteins, probably due to the accumulation of lipid peroxidation metabolites. This dual effect highlights the intricate balance and interactions within oxidative pathways in goats, which are affected by supplementation with *Curcuma longa* and *Rosmarinus officinalis* extract mixture. These observations are further supported by the correlation analysis between the control and experimental groups, which highlight more interdependencies in the experimental group. These findings contribute to a deeper understanding of how dietary interventions affect oxidative stress markers and biochemical pathways in PWI goats.

## 5. Conclusions

Supplementation with the turmeric–rosemary mixture reduced lipoperoxidation and increased TAC in the tested goat bucks. This may be attributed to the activation of antioxidant pathways and improved neutralization of ROS. However, the observed increase in OMPs may be due to increased levels of aldehyde and ketone derivatives, specifically OMP AD and OMP KD, produced by lipid peroxidation. These derivatives can alter protein structures and interfere with their biological functions.

The lack of changes in SOD, GPx, GR, CP, ALT, or AST activity, and lower CAT activity, suggest that supplementation effectively enhanced antioxidant defenses without adversely affecting liver enzyme activities: a favorable biochemical response.

Further studies are needed to determine the effect of different doses of the dry turmeric–rosemary mixture. These studies should examine other biochemical parameters in blood serum. The findings would provide a clearer indication of the dose that would improve health and not lead to increased levels of aldehyde and ketone derivatives.

## Figures and Tables

**Table 1:** Correlations between antioxidant and biochemical parameters in blood serum of young bucks after turmeric–rosemary supplementation.

GROUP		CONTROL										
	Parameter	TBAR	TAC	OMP AD	OMP KD	SOD	CAT	GR	GPx	CP	AST	ALT
TREATED	TBAR	―	0.246	−0.370	0.480	0.473	−0.202	−0.158	0.141	−0.370	−0.280	0.614 **
	TAC		―	0.473	0.114	0.074	−0.044	0.473	−0.243	−0.475	0.062	0.074
	OMP AD	0.203	−0.788 **	―	0.699	−0.337	−0.214	0.124	0.006	−0.338	−0.652 *	0.062
	OMP KD	0.108	0.744 **	0.673	―	−0.354	−0.130	−0.582	−0.280	0.228	0.308	−0.243
	SOD	0.308	0.108	0.480	0.354	―	−0.323	0.200	0.623	0.119	0.3045	0.354
	CAT	−0.302	−0.96	0.074	−0.095	−0.078	―	−0.194	−0.237	0.505	0.625	0.258
	GR	−0.243	0.213	0.335	0.626*	−0.456	0.246	―	0.200	0.819 **	0.074	−0.095
	GPx	0.354	−0.475	0.305	−0.095	0.354	−0.436	−0.390	―	0.119	0.764 **	0.228
	CP	−0.130	0.3022	−0.145	−0.456	−0.389	−0.624	0.063	−0.456	―	0.496	−0.370
	AST	0.480	−0.280	−0.475	0.203	0.074	0.458	−0.210	−0.475	0.302	―	0.952 **
	ALT	0.228	0.246	−0.478	−0.333	0.354	0.368	−0.221	−0.392	−0.211	0.946 **	―

OMP—lipid peroxidation and oxidative modification of proteins; TAC—total antioxidant capacity; SOD—super-oxide dismutase; CAT—catalase; GPx—glutathione peroxidase; GR—glutathione reductase; ALT—alanine aminotransferase; AST—aspartate aminotransferase; CP—ceruloplasmin. **—significance at *p* ≤ 0.01. *—significance at *p* ≤ 0.05. ―: no correlation (*p* > 0.05).

## Data Availability

The datasets used and/or analyzed during the current study are available from the corresponding author on reasonable request.
